# Sliding principal component and dynamic reward reinforcement learning based IIoT attack detection

**DOI:** 10.1038/s41598-023-46746-0

**Published:** 2023-11-27

**Authors:** Vijayan Ellappan, Anand Mahendran, Murali Subramanian, Jeevanandam Jotheeswaran, Adil O. Khadidos, Alaa O. Khadidos, Shitharth Selvarajan

**Affiliations:** 1 School of Information Technology and Engineering, VIT, Vellore, 632014 India; 2 School of Computer Science and Engineering, Vellore Institute of Technology, Vellore, India; 3 Executive Director - Technology Enabled Learning, Alliance University, Bangalore, India; 4 Department of Information Technology, Faculty of Computing and Information Technology, King Abdulaziz University, Jeddah, Saudi Arabia; 5 Department of Information Systems, Faculty of Computing and Information Technology, King Abdulaziz University, Jeddah, Saudi Arabia; 6 Director, Artificial Intelligent and Data Analysis Centre, King Abdulaziz University, 21589 Jeddah, Saudi Arabia; 7 Department of Computer Science, Kebri Dehar University, Kebri Dehar, Ethiopia; 8 School of Built Environment, Engineering and Computing, Leeds Beckett University, Leeds, LS1 3HE UK

**Keywords:** Engineering, Electrical and electronic engineering

## Abstract

The Internet of Things (IoT) involves the gathering of all those devices that connect to the Internet with the purpose of collecting and sharing data. The application of IoT in the different sectors, including health, industry has also picked up the threads to augment over the past few years. The IoT and, by integrity, the IIoT, are found to be highly susceptible to different types of threats and attacks owing to the networks nature that in turn leads to even poor outcomes (i.e., increasing error rate). Hence, it is critical to design attack detection systems that can provide the security of IIoT networks. To overcome this research work of IIoT attack detection in large amount of evolutions is failed to determine the certain attacks resulting in a minimum detection performance, reinforcement learning-based attack detection method called sliding principal component and dynamic reward reinforcement learning (SPC–DRRL) for detecting various IIoT network attacks is introduced. In the first stage of this research methodology, preprocessing of raw TON_IoT dataset is performed by employing min–max normalization scaling function to obtain normalized values with same scale. Next, with the processed sample data as output, to extract data from multi-sources (i.e., different service profiles from the dataset), a robust log likelihood sliding principal component-based feature extraction algorithm is applied with an arbitrary size sliding window to extract computationally-efficient features. Finally, dynamic reward reinforcement learning-based IIoT attack detection model is presented to control the error rate involved in the design. Here, with the design of dynamic reward function and introducing incident repository that not only generates the reward function in an arbitrary fashion but also stores the action results in the incident repository for the next training, therefore reducing the attack detection error rate. Moreover, an IIoT attack detection system based on SPC–DRRL is constructed. Finally, we verify the algorithm on the ToN_IoT dataset of University of New South Wales Australia. The experimental results show that the IIoT attack detection time and overhead along with the error rate are reduced considerably with higher accuracy than that of traditional reinforcement learning methods.

## Introduction

The Industrial Internet of Things (IIoT) is an immense organization comprising of several perceptive associated instruments that recommend several dominances to intelligent computing in organizations, ranging between productions and services. With the fourth industrial insurgence, manufacturing and industrial techniques and viewpoints pick up the threads to be automated with modernized technology. Moreover, the Internet of Things (IoT) and communications between machines are consolidated to improve automation, enhance communications and evolve machines without the requirement for human interaction. Owing to the reason that tremendous sensors and devices are associated to generate several data, acquiring data in an accurate manner, processing them and transmission of the respective data in a safe manner become analytic in IIoT platforms. With the emergence of IIoT, diversity and complications are said to persist as far as cyber-attacks are concerned, making the prevailing anomaly detection methods less efficient to function. An ensemble deep learning method called, deep long short-term memory (LSTM) and auto-encoder (AE) method was proposed in^1^ with the objective of identifying out-of-norm activities for cyber threat hunting in IIoT. Here, the LSTM was applied for creating past and present data for accessing normal data patterns and minimizing dimension via AE. Also, the issues concerning imbalanced nature of IIoT datasets were addressed, therefore improving accuracy, precision, recall and training time. However, it failed to focus on IIoT detection comprised attack detection time and overhead. To address on these two factors, in this work, normalized scaling is first performed with the raw dataset and then pertinent information among the processed input features with minimum informational loss is obtained via log likelihood sliding window and principal component functions. Deep learning and big data analytics have considerable prospective in crafting and evolving vigorous security methods for IIoT networks. In^2^, a novel hybrid deep random neural network (HDRaNN) for detecting cyber attack in IIoT was presented. Here, the deep random neural network was integrated with multilayer perceptron and dropout regularization with which 16 distinct types of cyber attacks were detected, therefore improving precision, accuracy, recall and F1-score significantly. Though several performance factors like, precision, accuracy, recall and F1-score were improved. However, the error rate and overhead involved during detecting cyber attack in IIoT was not focused. To address on this issue, dynamic reward reinforcement learning-based IIoT attack detection model is designed. With this design mechanism, a dynamic reward function is introduced that according to the service profiles, detects the attack in a timely manner. Moreover, by storing the results in the incident repository, the overhead involved in detecting cyber attack will also be improved to a greater extent.

Several researchers are now concerned in including a pinnacle extent of security to IIoT. Machine learning (ML) methods were utilized for building a pinnacle extent of security potentialities on the basis of intrusion detection systems (IDSs). In^3^, ML methods were applied to realistic dataset called ToN-IoT from large-scale, heterogeneous IoT network and was tested in both binary and multi-class classification problems. In^4^, state-of-the-art intrusion detection systems (IDS) were surveyed. In addition, hybrid IDS architecture was also introduced via machine learning method to focus on the accuracy aspect. However, this consistency also instigates IoT with a pervasive array of essential security threats that necessitates significant issues to be saturated. In^5^, deep learning (DL) driven software defined networking (SDN) enabled IDS was proposed with the objective of combating against cyber threats in IoT communications. The Industrial Internet of Things (IIoT), over the past few years have instigated a revolution both in the domain of production and manufacturing sectors by automating production management with minimal human effort. In spite of sizeable amount of evolutions in IIoT attack detection. However, it failed to detect the certain attacks resulting in a low detection performance. To address on this aspect, a deep learning-based two level network intrusion detection system (DL-TL-NIDS) was presented in^6^ for IIoT environment. In^7^, two novel mechanisms for selecting adversarial samples to retrain a classifier was proposed based on two distinct factors, distance and probability distribution. The first one was based on the distance from cluster center and the second probability distribution was employed on the kernel learning for industrial IoT detection. One of the IIoT influencing evaluative security concerns is the false data injection attack. However, it failed to improve the precision. Here, the FDI attacks deceive the industrial manifestos by counterfeiting their sensor assessments. In^8^, a novel auto encoders (AE) method for detecting FDI attack was presented. Here, the association of data between time and space was utilized that in turn assisted in identifying falsified data. This paper proposes a computationally-efficient and robust reinforcement learning-based attack detection method called, sliding principal component and dynamic reward reinforcement learning (SPC–DRRL) to detecting IIoT attacks. It provides a solution towards detection of IIoT attacks. The IIoT detection time and accuracy improves by normalizing and scaling the raw data for obtaining computationally efficient features to be extracted. Secondly, it aims to decrease the IIoT attack detection error rate and overhead by not only putting the resultant samples in the incident repository but also introducing loss function via dynamic reward to therefore ensure robust attack detection. The main pertinent contributions of this article are summarized as follows.

- A sliding principal component and dynamic reward reinforcement learning (SPC–DRRL) is introduced to reinforcement learning-based attack detection method to ensure security that in turn maximizes the number of correctly detectable classes in a timely manner.
- A log likelihood sliding principal component-based feature extraction algorithm for extracting data from multi-sources by using new feature extraction model.
- A dynamic reward reinforcement learning for controlling error rate by a novel IIoT attack detection model using dynamic reward function and introducing incident repository error rate.
- We perform various simulations using TON_IoT dataset to evaluate and validate the performance of the proposed method and compare it with the existing and state-of-the-art methods.

The rest of this article is organized as follows. Section “Related works” provides a summary of the relevant work carried out in the domain of IIoT attack detection. Section “ToN-IoT dataset description” provides the dataset description in use. In “Methodology” section the overall framework of the proposed method is presented. In “Simulation analysis” section both the qualitative and quantitative analysis for the proposed IIoT attack detection method is investigated. Also experimental results are presented in this section. Finally, “Conclusion” section concludes this article.

## Related works

The IIoT is influencing the IoT technology and utilizing IoT technology improves the network intelligence in optimization and automation of industrial processes. However, the utilization of the IoT though enhances connectivity with corporate networks, but introduced the probability of cyber-attacks against these systems. In^9^, a novel machine learning algorithm was introduced to ease the class imbalance issue by measuring an optimized weight for each machine learning-based decision. With this, high detection rate and low false alarm rate were ensured. An elaborative study on federated deep learning methods for IIoT was investigated in^10^. Also a review of vulnerabilities concerning security and privacy were also discussed here. Malicious traffic identification employing deep learning mechanisms has made an appearance as a pivotal element of IDS. Recurrent neural network based IDS for binary and multiclass classification was designed in^11^ that in turn not only ensured precision but also ensured accuracy to a greater extent.

An extensive degree of data processing takes place at the cloud to execute different types of analytics in IIoT. To cope with the analytics utilizing such an enormous amount of IIoT data, several deep learning based analytical methods are employed. The learning process has to act in accordance with the reliability and trustworthy life cycle for critical analysis and decision making. In a similar manner, taking into consideration the susceptibilities in several aspects of an IIoT network are also not said to be avoided. A survey of machine and deep learning for attack detection in IIoT was investigated in^12^. A holistic present day IoT IDS and survey of materials, methods, validation techniques for constructing IIoT IDS was presented in^13^. In^14^, a comprehensive survey on threats concerning security and measures taken to handle the threats employing artificial intelligence based mechanisms were discussed. A reliable routing attack based IIoT attack detection mechanism was proposed in^15^ by introducing generative adversarial neural classifier. With this type of classifier ensures centralized attack detection. In^16^, a topological and flow feature-based deep learning method (GLD-Net) was proposed with the objective of extracting the topological features and also employed graph attention network (GAT) for obtaining correlations between non-Euclidean features. Owing to this the average detection accuracy was said to be improved. A novel anomaly-based intrusion detection employing convolutional neural network model was presented in^17^ that in turn created multiclass classification therefore ensuring high accuracy and precision. A two-phase anomaly detection model employing ensemble classification was proposed in^18^. Ensemble blending using random forest technique was employed for efficient prediction of class labels. Followed by which Adam optimizer was employed for ensuring accuracy prediction. Multilayer deep learning techniques were employed in^19^ for detecting botnet attacks in IIoT. A trust-based hybrid cooperative RPL protocol (THC-RPL) was presented in^20^ with the objective of detecting malicious Sybil nodes in routing protocol for low power and lossy protocol based IoT network. But, the storage cost was not improved. An Artifcial Intelligence-based Lightweight Blockchain Security Model (AILBSM) designed in^21^ to secure the privacy and security of IIoT systems by using AI mechanisms with simplified and improved security operations. However, the time consumption was not improved. In^22^, AI-based and device algorithms are also examined to attain a more effective IoT process namely AIoT, combined with Internet and artificial intelligence. But, minimize the reaction times and increased reliability. The intrusion detection system (IDS) was designed in^21^ to monitors the network events and filters the abnormal activities. In^24^, networks intrusion detection system (NIDS) method was developed into existing methods that mainly focus on identify the intrusions from datasets with aid of classification methods. Also, the improve the detection accuracy and predicted outcomes.

### Motivation

The motivation of this proposed work is IIoT attack detection based reinforcement learning to assure the securities which turn better the number of correctly detectable classes. The IIoT attack detection in large amount of evolutions is failed to employ the certain attacks resulting in a lesser detection performance, reinforcement learning-based attack detection method determined for detecting different IIoT network attacks. At first, preprocessing is determine the normalized values with same scale. Next, with the processed sample data as output is to extract data from multi-sources. At last, IIoT attack detection model is performed to control error rate involved. Here, with reducing the attack detection error rate. To make industrial intrusion detection more advanced, a combination of the abovementioned industrial intrusion detection methods called, sliding principal component and dynamic reward reinforcement learning (SPC–DRRL) is proposed. Each method has its owing specific advantages and hence to safeguard the IIoT network from different attacks, sliding principal component-based feature extraction and dynamic reward reinforcement learning-based classification for detecting IIoT attack is presented. The elaborate description of the (SPC–DRRL) method is provided in the following subsections.

## ToN-IoT dataset description

The TON_IoT datasets is considered to be one of the new generations of Industry 4.0/Internet of Things (IoT) and Industrial IoT (IIoT) datasets for validating the exactness and significance of distinct cyber security applications on the basis of artificial intelligence (AI), i.e., machine learning and deep learning algorithm. The datasets have been referred to as ‘ToN_IoT’ as they consist of heterogeneous data sources obtained from Telemetry datasets of IoT and IIoT sensors. The datasets were obtained from large-scale network created at the Cyber Range and IoT Labs, the School of Engineering and Information technology (SEIT), UNSW Canberra @ the Australian Defence Force Academy (ADFA). Moreover, seven profiles namely, IoT fridge activity including six features (i.e., date, time, fridge_temperature, temperature_condition, label_condition and type), IoT garage activity including six features (i.e., date, time, door_state, sphone_signals, label and type), IoT GPS_tracker activity including six features (i.e., date, time, latitude, longitude, label and type), IoT Modbus activity including seven features (i.e., date, time, FC1_Read_Input_Register, FC2_Read_Discrete_Value, FC3_Read_Holding_Register, FC4_Read_Coil, label and type), IoT Motion_Light activity including six feuatres (i.e., date, time, motion_status, light_status, label and type), IoT Thermostat activity including six features (i.e., date, time, current_temperature, thermostat_status, label and type) and IoT Weather activity including seven features (i.e., date, time, temperature, pressure, humidity, label and type) were included for validating and testing various attack detection in IIoT.

## Methodology

**Figure 1:**
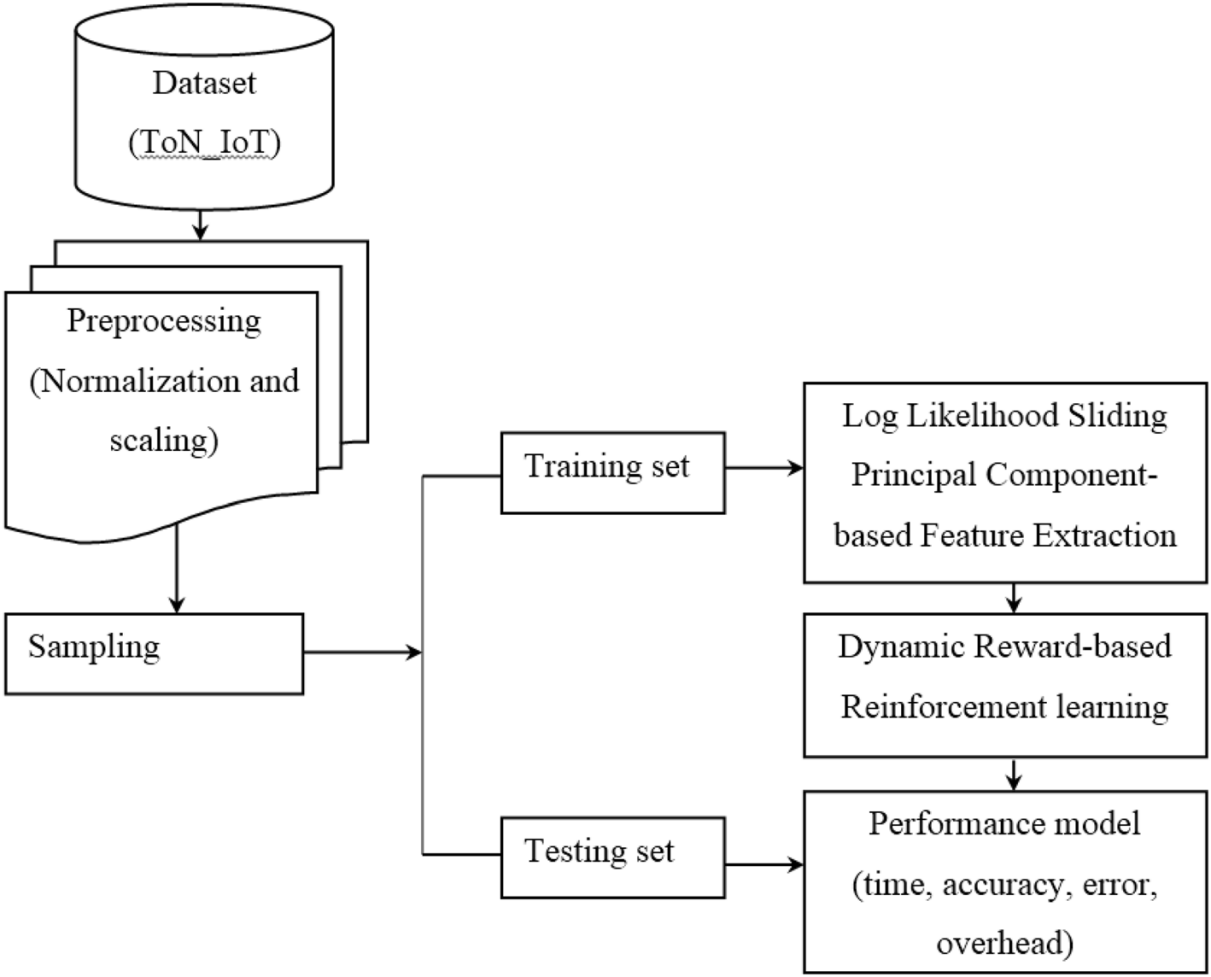
Block diagram of sliding principal component and dynamic reward reinforcement learning (SPC–DRRL).

The architecture of the proposed sliding principal component and dynamic reward reinforcement learning (SPC–DRRL) for detecting various IIoT network attacks method is depicted in Fig. 1, whereby there are three main phases, namely, the pre-processing phase, the feature selection phase, and the classification phases.

As shown in the above figure, in the pre-processing phase, we load the TON_IoT Dataset (training set, validation set, and testing sets). The feature values in the dataset are cleaned and normalized employing min–max normalization scaling-based preprocessing algorithm. In the feature extraction phase, the normalized and scaled training dataset is utilized to compute the dimensionality reduced features using the log likelihood sliding principal component-based feature extraction algorithm. Finally, dynamic reward reinforcement learning-based IIoT attack detection model is designed using the ToN_IoT dataset. The building blocks of the proposed sliding principal component and dynamic reward reinforcement learning (SPC–DRRL) for detecting various IIoT network attacks are explained in more detail in the next subsections.

### Min–max normalization scaling based preprocessing

Data preprocessing remains the first step for attack detection in IIoT after the acquisition and loading of the TON_IoTdataset. Data preprocessing is very essential as it assists in discarding outliers and eliminating unnecessary attributes. It is calculated to transpose the raw network data stored in the form of vector to a format that is significant to utilization for further analysis. With the presence of seven distinct service profiles present in the dataset, seven different input vectors are formulated as given below (with different numbers of rows represented in the form of ‘$i$’ and columns represented in the form of ‘$j$’ for each vector).

1 $$FV=\left[\begin{array}{cccc}{FV}_{1}{IF}_{1}& {FV}_{1}{IF}_{2}& \dots & {FV}_{1}{IF}_{j}\\ {FV}_{2I}{F}_{1}& {FV}_{2}{IF}_{2}& \dots & {FV}_{2}I{F}_{j}\\ \dots & \dots & \dots & \dots \\ {FV}_{i}{IF}_{1}& {FV}_{i}{IF}_{2}& \dots & {FV}_{i}{IF}_{j}\end{array}\right],\; where \; i=59945, j=7$$

2 $$GD=\left[\begin{array}{cccc}{GD}_{1}I{F}_{1}& {GD}_{1}{IF}_{2}& \dots & {GD}_{1}{IF}_{j}\\ {GD}_{2}{IF}_{1}& {GD}_{2}{IF}_{2}& \dots & {GD}_{2}{IF}_{j}\\ \dots & \dots & \dots & \dots \\ {GD}_{i}{IF}_{1}& {GD}_{i}{IF}_{2}& \dots & {GD}_{i}{IF}_{j}\end{array}\right],\; where \; i=59588, j=7$$

3 $$GT=\left[\begin{array}{cccc}{GT}_{1}{IF}_{1}& {GT}_{1}{IF}_{2}& \dots & {GT}_{1}{IF}_{j}\\ {GT}_{2}{IF}_{1}& {GT}_{2}I{F}_{2}& \dots & {GT}_{2}{IF}_{j}\\ \dots & \dots & \dots & \dots \\ {GT}_{i}{IF}_{1}& {GT}_{i}{IF}_{2}& \dots & {GT}_{i}{IF}_{j}\end{array}\right],\; where \; i=58961, j=7$$

4 $$MB=\left[\begin{array}{cccc}{MB}_{1}{IF}_{1}& {MB}_{1}{IF}_{2}& \dots & {MB}_{1}{IF}_{j}\\ {MB}_{2}I{F}_{1}& {MB}_{2}{IF}_{2}& \dots & {MB}_{2}I{F}_{j}\\ \dots & \dots & \dots & \dots \\ {MB}_{i}I{F}_{1}& {MB}_{i}{IF}_{2}& \dots & {MB}_{i}{IF}_{j}\end{array}\right],\; where \; i=51107, j=9$$

5 $$ML=\left[\begin{array}{cccc}{ML}_{1}{IF}_{1}& {ML}_{1}{IF}_{2}& \dots & {ML}_{1}{IF}_{j}\\ {ML}_{2I}{F}_{1}& {ML}_{2}{IF}_{2}& \dots & {ML}_{2}{IF}_{j}\\ \dots & \dots & \dots & \dots \\ {ML}_{i}{IF}_{1}& {ML}_{i}{IF}_{2}& \dots & {ML}_{i}{IF}_{j}\end{array}\right],\; where \; i=59489, j=7$$

6 $$TS=\left[\begin{array}{cccc}{TS}_{1}{IF}_{1}& {TS}_{1}{IF}_{2}& \dots & {TS}_{1}{IF}_{j}\\ {TS}_{2}{IF}_{1}& {TS}_{2}{IF}_{2}& \dots & {TS}_{2}{IF}_{j}\\ \dots & \dots & \dots & \dots \\ {TS}_{i}{IF}_{1}& {TS}_{i}{IF}_{2}& \dots & {TS}_{i}{IF}_{j}\end{array}\right],\; where \; i=52775, j=7$$

7 $$W=\left[\begin{array}{cccc}{W}_{1}{IF}_{1}& {W}_{1}{IF}_{2}& \dots & {W}_{1}{IF}_{j}\\ {W}_{2}{IF}_{1}& {W}_{2}{IF}_{2}& \dots & {W}_{2}{IF}_{j}\\ \dots & \dots & \dots & \dots \\ {W}_{i}{IF}_{1}& {W}_{i}{IF}_{2}& \dots & {W}_{i}{IF}_{j}\end{array}\right],\; where \; i=59261, j=8$$

**Figure 2:**
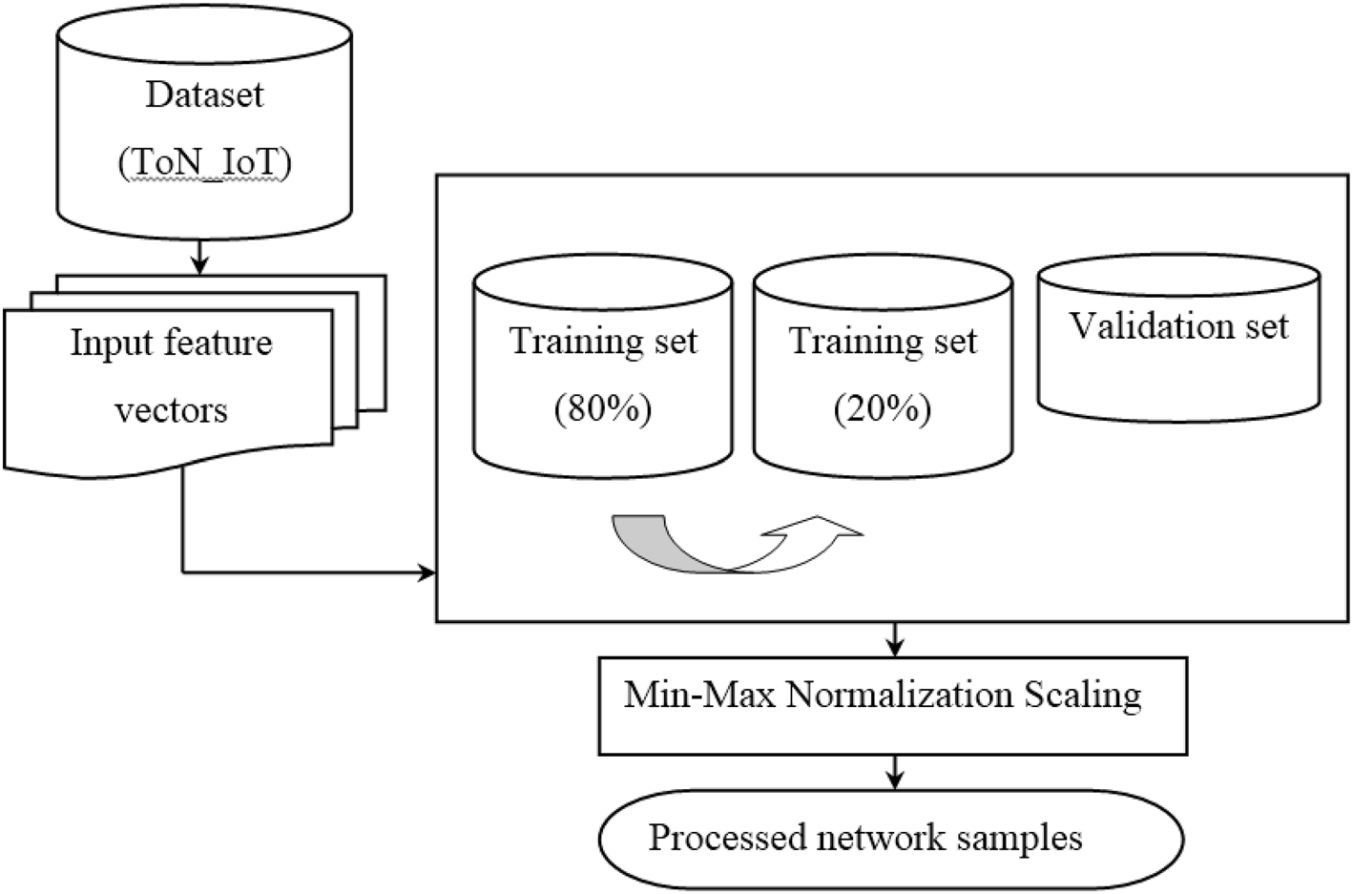
Structure of min–max normalization scaling-based preprocessing.

With the above input feature vector values, in our work min–max normalization scaling function is utilized to have all the feature vector values with a-like scale. Figure 2 shows the structure of min–max normalization scaling-based preprocessing model.

As illustrated in the above figure, let us consider training subset of input feature vector ‘$A=\left\{\left({FV}_{i},{FV}_{j}\right), \left({GD}_{i},{GD}_{j}\right), \left({GT}_{i},{GT}_{j}\right), \left({MB}_{i},{MB}_{j}\right), \left({ML}_{i},{ML}_{j}\right), \left({TS}_{i},{TS}_{j}\right), \;and \;\left({W}_{i},{W}_{j}\right)\right\}$’ respectively that are selected arbitrarily given dataset ‘$DS=\left(A,B\right)$’ with ‘$B$’ denoting the classifier output. Then, the min–max normalization scaling function normalizes the attributes or the vector feature values in the range of ‘$\left[\mathrm{0,1}\right]$’ as given below.

8 $$NIF=\left(A-B\right)\frac{{IF}_{n}-Min\left({IF}_{n}\right)}{Max\left({IF}_{n}\right)-Min\left(I{F}_{n}\right)}$$

From the above Eq. (8), the normalized scaling results of each feature vector ‘$I{F}_{NS}$’ is obtained based on the minimum ‘$Min\left({IF}_{n}\right)$’ and maximum ‘$Max\left({IF}_{n}\right)$’ values of the feature vector of concern. This min–max normalization scaling function acts as a protecting mechanism by eliminating the values of each feature within an explicit range. The pseudo code representation of min–max normalization scaling-based Preprocessing is given below.

**Algorithm 1:**
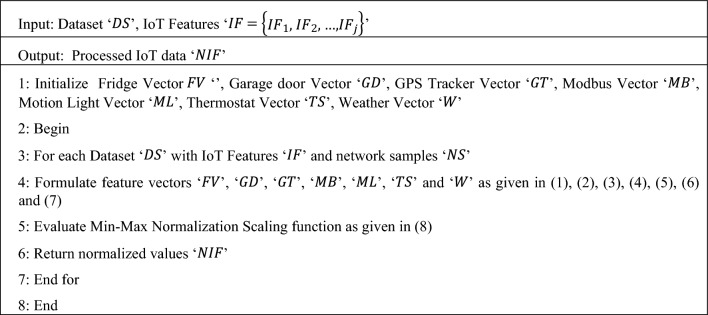
Min-Max Normalization Scaling

In Algorithm 1, describe the aim of discarding outliers and eliminating unnecessary attributes. At first, the raw dataset is modeled into distinct vectors. Initialize the seven distinct service profiles perform the dataset and seven different input vectors. After that for each vector, min–max normalization scaling function is applied to obtain the normalized values in the range ‘$\left[\mathrm{0,1}\right]$’ so that all the values of the attributes or features possess same scale. The normalized scaling results of each feature vector are obtained based on the minimum and maximum values of the feature vector of concern. This min–max normalization scaling function acts as a protecting mechanism by eliminating the values of each feature within an explicit range. This in turn makes further processing simpler both in terms of time and accuracy.

### Log likelihood sliding principal component-based feature extraction

**Figure 3:**
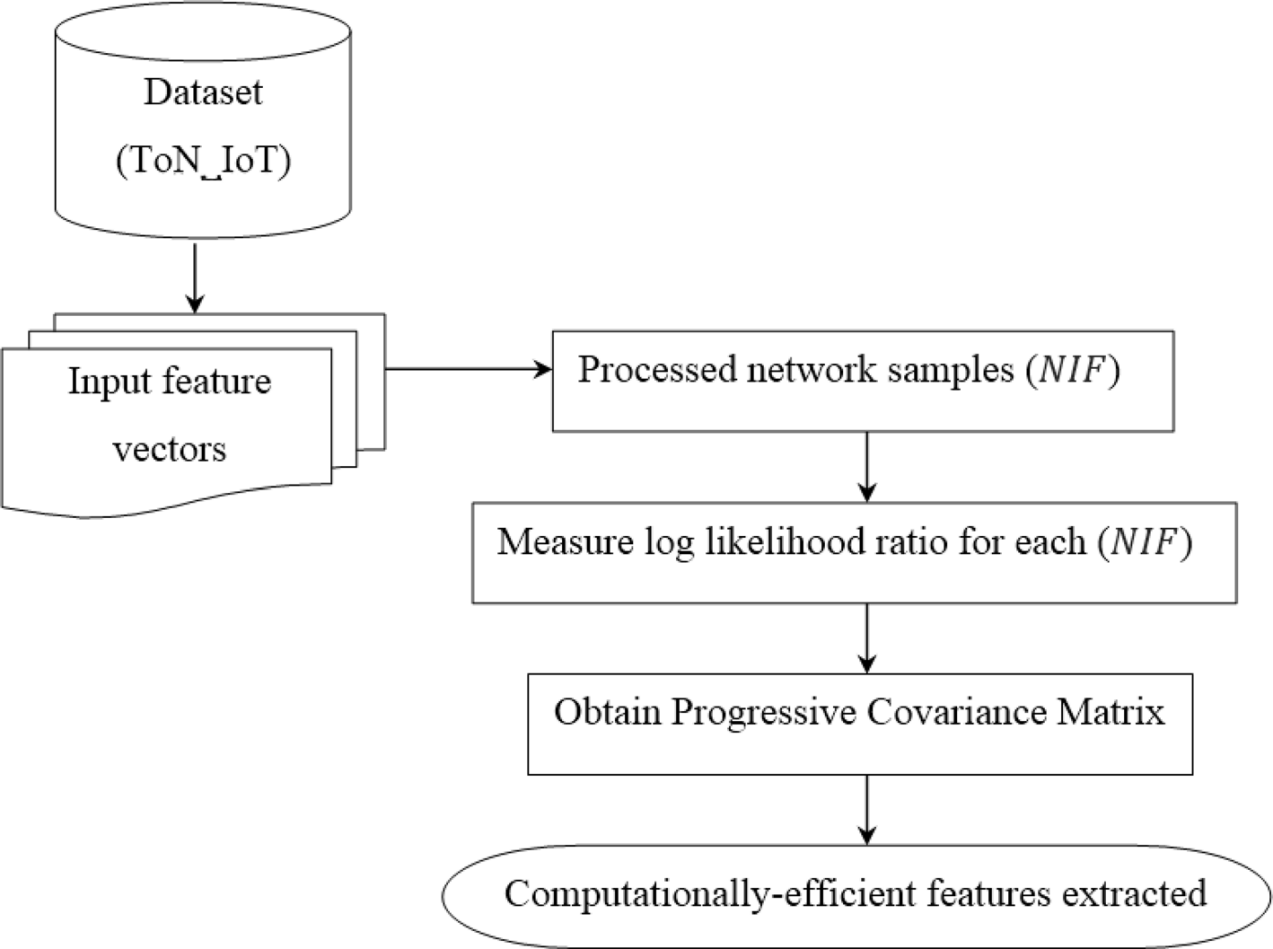
Structure of log likelihood sliding principal component-based feature extraction.

Once the raw IIoT dataset are processed or preprocessed step has been carried out, the second step in IIoT attack detection is the relevant feature extraction. Feature extraction is considered as yet another important process in IIoT attack detection because not all the features are required for IIoT attack detection. By performing feature extraction not only the feature dimensionality reduction is said to be achieved but also extracts pertinent information among the raw input features with minimum informational loss too. IoT data related to industrial applications has the characteristics consisting of both normal and seven attacks, multi-sources (i.e., obtained from seven distinct service profiles), conventional feature extraction models are inadequate of encountering real-time demands. To address on this aspect, a Log Likelihood sliding principal component-based feature extraction is designed. Here, by employing the log likelihood ratio for the corresponding sliding window, principal components are extracted. This is owing to the reason that the dataset employed in our work possesses different service profiles and also relevant feature for each service profile differs. Figure 3 shows the structure of Log Likelihood sliding principal component-based feature extraction model.

As illustrated in the above figure, the log likelihood sliding principal component-based feature extraction model extracts features on the basis of statistical functions. It identifies the eigen vectors possessing highest eigen values in the progressive covariance matrix with arbitrary length sliding window. The arbitrary length sliding window will remain in the fixed length state until a new service profile is detected or the current service profile is terminated. After the end of service profile is detected, the window will either sequentially dilates, discarding all the reorganized features or it will sequentially dilates, continuing from its reorganized feature size. In both cases features or attributes in charge for the current change point are discarded. The remaining extracted features called as principal components that reduce dimensionality without losing much information. Let us consider the processed IoT data points be ‘${NIF}_{i}=\left\{{NIF}_{1i}, {NIF}_{2i}\dots ,{NIF}_{Mi}\right\}$’ and put these vectors into matrix. Then, the processed IoT data points are centered in such a manner so as to subtract off the mean of each column as given below.

9 $${NIF}_{i,b}={NIF}_{i,a}-\mu$$

From the above Eq. (9), the mean value ‘$\upmu$’, is subtracted from each attribute ‘${\mathrm{NIF}}_{\mathrm{i},\mathrm{a}}$’ and storing the result as ‘${\mathrm{NIF}}_{\mathrm{i},\mathrm{b}}$’. Let us further assume that an arbitrary process ‘$\mathrm{NIF}$’ is sampled at a fixed time interval ‘$\mathrm{t}$’ forming a sequential observation ‘$\mathrm{NIF}\left(\mathrm{t}\right)$’. Upon successful completion of iteration decision is made to infer whether or not there is a transformation in process (i.e., transformation between distinct service profiles) evolving in a change point. The test for change at time ‘$t0$’ from observations ‘${O}_{i}$’ and ‘${O}_{k}$’ is based on log likelihood ratio as given below.

10 $${NIF}_{n}=\sum_{i=1}^{n}{nif}_{i}=\mathrm{ln}\frac{{Prob}_{\theta 1}\left({O}_{i}\right)}{{Prob}_{\theta 0}\left({O}_{i}\right)}$$

Next, according to the transformation, the progressive covariance matrix is evaluated as given below.

11 $$Cov\left(n\right)=\frac{1}{n}\sum_{i=1}^{n}{NIF}_{i}{\left(NIF\right)}_{i}^{T}\frac{{Cov}_{i-1}}{\left|{Cov}_{i-1}\right|}$$

With the above progressive covariance matrix results ‘$Cov\left(n\right)$’, the eigen vector ‘$V$’ and eigen value results ‘$D$’ are obtained as given below.

12 $${V}^{-1}CovV=D$$

Finally, the eigen values less than ‘$\eta$’ is rejected whereas the other features are selected, therefore minimizing the dimension of data. The pseudo code representation of log likelihood sliding principal component-based feature extraction is given below.

**Algorithm 2:**
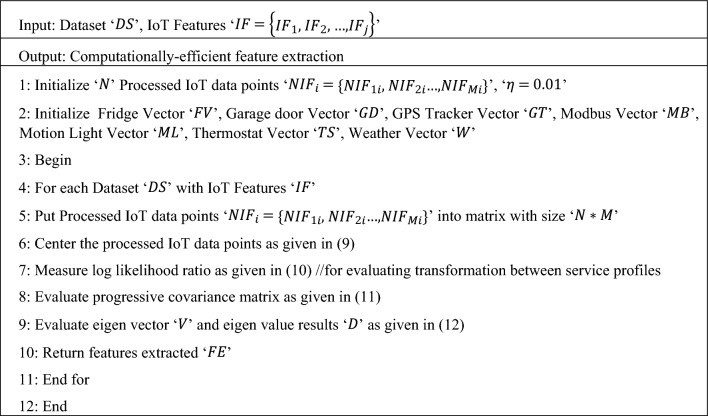
Log Likelihood Sliding Principal Component-based Feature Extraction

From Algorithm 2, the log likelihood sliding principal component-based feature extraction algorithm represent with normalized and scaled results provided as input, the objective remains the extracted computationally-efficient features. With this objective, transformation between service profiles is performed using the log likelihood ratio. Next, for each service profiles progressive covariance matrix is formulated. Finally, with the progressive covariance results, pertinent features were extracted in a computationally efficient manner.

### Dynamic reward reinforcement learning-based IIoT attack detection

**Figure 4:**
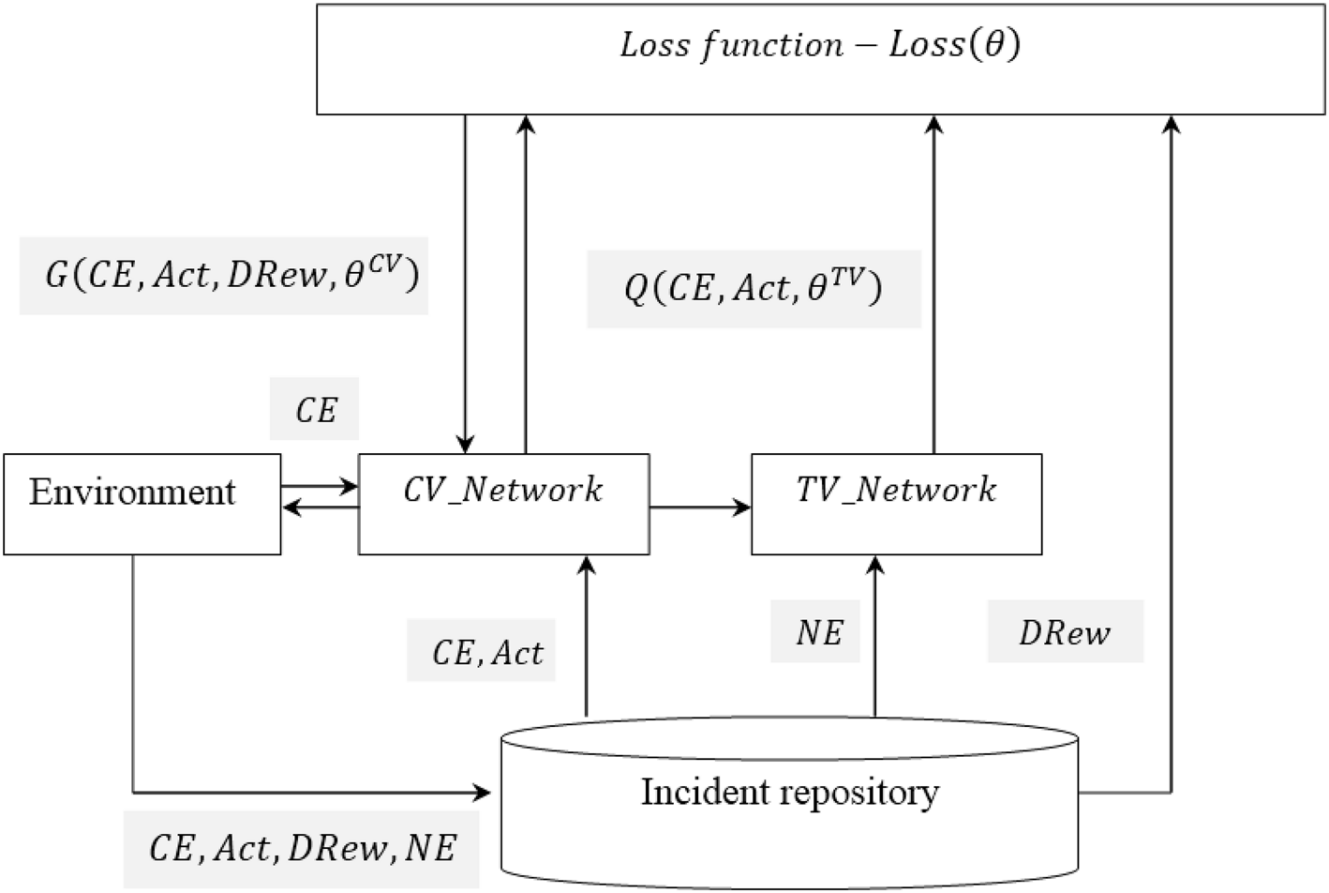
Structure of dynamic reward reinforcement learning-based IIoT attack detection.

In the open network environment, industrial control systems face huge security risks and is hence said to be highly susceptible to network attacks. The prevailing attack detection methods of industrial control networks have the issue of a modest and flexible detection in the presence of distinct service profiles. To address on this aspect, in this work a dynamic reward reinforcement learning-based IIoT attack detection model is presented and builds a learning framework with continuous learning potentiality. The dynamic reward reinforcement learning-based IIoT attack detection is specifically composed of two elements, an agent and environment. Here, the agent constantly communicates with the environment, produces an action via the ‘$Q$’ function, then performs the action and enters a new environment. The dynamic reward reinforcement learning-based IIoT attack detection model will reward the agent on the basis of the actions carried out by the agent. The agent makes decisions by maximizing rewards in a dynamic fashion. Figure 4 shows the structure of dynamic reward reinforcement learning-based IIoT attack detection model.

From the above figure, ‘$CE$’ refers to the current environment, ‘$NE$’ refers to the next environment, ‘$Act$’ denotes the action performed under the current environment via ‘$Q$’ function and ‘$DRew$’ denotes the dynamic reward gained by performing action under the current environment respectively. Here, the action selection is done according to greedy strategy, which refers to how likely the current sampling is to make decisions based on the dynamic reward via ‘$Q$’ function generated by current training network. To start with the dynamic reward is formulated as given below.

13 $$DRew=FE\left[\frac{Rew\left[AD\right]+Rew\left[AA\right]}{No\_of\left[A\right]}\right]$$

From the above Eq. (13), dynamic reward ‘$DRew$’ is measured based on the agents reward upon successful detecting of attack ‘$Rew\left[AD\right]$’, agents reward when attacker is attacked ‘$Rew\left[AA\right]$’ with respect to the total numbers of attacks ‘$No\_of\left[A\right]$’ in a simulation settings. Next, the loss function ‘$Loss\left(\theta \right)$’ of dynamic reward reinforcement learning-based IIoT attack detection model referring to the timing different between current network value and target network value is mathematically formulated as given below.

14 $$Loss\left(\theta \right)=E\left[\left(G\left(CE,Act, DRew,{\theta }^{CV}\right)-Q\left(CE, Act,{\theta }^{TV}\right)\right)\right]$$

From the above Eq. (14), the loss function results ‘$Loss\left(\theta \right)$’ is obtained based on the result of restoring the placement of the action ‘$Act$’ in ‘$Q\left(CE, Act,{\theta }^{TV}\right)$’ with the Dynamic Reward ‘$DRew$’ respectively (i.e., ‘${\theta }^{CV}$’ denoting the current parameter value and ‘${\theta }^{TV}$’ denoting the target parameter value). Finally, the network traffic is specifically split into normal and attack detection. Hence, there exist only two actions ‘$Act$’ in the dynamic reward reinforcement learning-based IIoT attack detection model. The mathematical formula of the action is represented as given below.

15 $$Act = \left\{ {\begin{array}{*{20}l} {0,} & {normal} \\ {1,} & {attack\;detection} \\ \end{array} } \right.$$

Based on the above resultant values from ‘$Act$’, the dynamic reward reinforcement learning-based IIoT attack detection model passes the network traffic to proceed with communication is the action is normal. On contrary, if the network traffic is malicious, dynamic reward reinforcement learning-based IIoT attack detection model will intercept the data and notes the attack type. Moreover, the resultant samples (i.e., attack or normal) are put into the incident repository for the next training. The pseudo code representation of Dynamic Reward Reinforcement Learning-based IIoT attack detection is given below.

**Algorithm 3:**
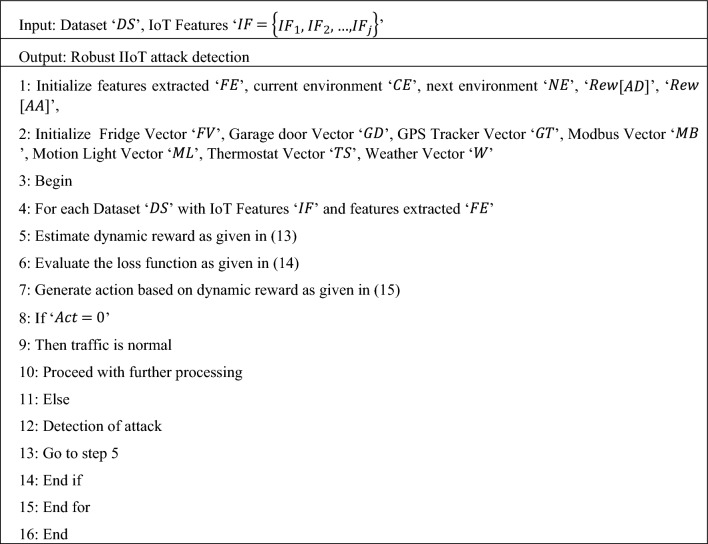
Dynamic Reward Reinforcement Learning-based IIoT attack detection

In Algorithm 3, describe the dynamic reward reinforcement learning-based IIoT attack detection algorithm with extracted features as input, dynamic reward is initially formulated. Followed by which, loss function is generated based on the difference between the current network value and target network value. Finally, the action is evaluated for detecting either the presence or absence of attack in IIoT network. Upon the presence of attack or if the resultant value of the action is ‘$1$’, conditional checking is made for each service profiles. For example in case of service profile (IoT Fridge activity), with the condition of temperature associated to the network, on the basis of threshold value (i.e., between 1.5 and 3.8—Ddos attack, between 3.9 and 5—backdoor, between 5.2 and 8—injection, between 8.2 and 12—password, between 12.2 and 15—ransomware, between 15.2 and 17—xss) different types of attacks are detected. With this the IIoT attack detection overhead and error rate are said to be reduced significantly.

## Simulation analysis

In this section, experiment is performed to validate the efficiency of the sliding principal component and dynamic reward reinforcement learning (SPC–DRRL) for detecting various IIoT network attacks using the experiment data that are available and accessible online from ToN_IoT dataset. Simulations are performed on a computer with an Intel(R) Core(TM) i5-7200 CPU @2.50GHz and 8.00GB of RAM. Comparative analysis is made with the two existing methods, Deep LSTM AE^1^, HDRaNN^2^ and state-of-the-art method, machine learning^3^ in terms of IIoT attack detection time, IIoT attack detection accuracy, IIoT attack detection overhead and IIoT attack detection error rate in Python high-level programming language.

### Qualitative analysis of SPC–DRRL

In this section the qualitative analysis of SPC–DRRL is discussed in detail. With the ToN_IoT dataset obtained as input, 20 network samples from service profile—IoT_Fridge is used for simulation as given below in Table 1.

**Table 1:** Network samples from IoT_Fridge service profile [ToN_IoT dataset].

ts	Date	Time	Fridge_temperature	Temp_condition	Label	Type
1556245200	25-Apr-19	19:20:00	11.55	high	1	ddos
1556245200	25-Apr-19	19:20:00	13.4	high	1	ddos
1556245205	25-Apr-19	19:20:05	1.75	low	1	ddos
1556459978	28-Apr-19	06:59:38	3.2	low	1	backdoor
1556459983	28-Apr-19	06:59:43	4	low	1	backdoor
1556459988	28-Apr-19	06:59:48	4.65	low	1	backdoor
1556209441	25-Apr-19	09:24:01	8.65	high	1	injection
1556209442	25-Apr-19	09:24:02	9.1	high	1	injection
1556209442	25-Apr-19	09:24:02	11.55	high	1	injection
1554061012	31-Mar-19	12:36:52	13.1	high	0	normal
1554061013	31-Mar-19	12:36:53	8.65	high	0	normal
1554061014	31-Mar-19	12:36:54	2	low	0	normal
1556327188	26-Apr-19	18:06:28	4.95	low	1	password
1556327189	26-Apr-19	18:06:29	13.25	high	1	password
1556327189	26-Apr-19	18:06:29	3	low	1	password
1556448879	28-Apr-19	03:54:39	4	low	1	ransomware
1556448884	28-Apr-19	03:54:44	1	low	1	ransomware
1556448889	28-Apr-19	03:54:49	7.7	high	1	ransomware
1556367221	27-Apr-19	05:13:41	4.05	low	1	xss
1556367225	27-Apr-19	05:13:45	2.75	Low	1	xss

With the above network samples obtained as input, first, seven different input vectors are formulated (with different numbers of rows represented in the form of ‘$i$’ and columns represented in the form of ‘$j$’ for each vector). In this work for performing simulation, the input vector for service profile—IoT_Fridge is formulated as given below.

$$FV=\left[\begin{array}{cccccc}1556245200& 25-\mathrm{Apr}-19& 19:20:00 & 11.55& high& 1\\ 1556245200& 25-\mathrm{Apr}-19& 19:20:00& 13.4& high& 1\\ 1556245205& 25-\mathrm{Apr}-19& 19:20:05 & 1.75& low& 1\\ 1556459978& 28-\mathrm{Apr}-19& 06:59:38 & 3.2& low& 1\\ 1556459983& 28-\mathrm{Apr}-19& 06:59:43 & 4& low& 1\\ 1556459988& 28-\mathrm{Apr}-19& 06:59:48 & 4.65& low& 1\\ 1556209441& 25-\mathrm{Apr}-19& 09:24:01 & 8.65& high& 1\\ 1556209442& 25-\mathrm{Apr}-19& 09:24:02 & 9.1& high& 1\\ 1556209442& 25-\mathrm{Apr}-19& 09:24:02 & 11.55& high& 1\\ 1554061012& 31-\mathrm{Mar}-19& 12:36:52 & 13.1& high& 0\\ 1554061013& 31-\mathrm{Mar}-19& 12:36:53 & 8.65& high& 0\\ 1554061014& 31-\mathrm{Mar}-19& 12:36:54 & 2& low& 0\\ 1556327188& 26-\mathrm{Apr}-19& 18:06:28& 4.95& low& 1\\ 1556327189& 26-\mathrm{Apr}-19& 18:06:29& 13.25& high& 1\\ 1556327189& 26-\mathrm{Apr}-19& 18:06:29& 3& low& 1\\ 1556448879& 28-\mathrm{Apr}-19& 03:54:39 & 4& low& 1\\ 1556448884& 28-\mathrm{Apr}-19& 03:54:44 & 1& low& 1\\ 1556448889& 28-\mathrm{Apr}-19& 03:54:49 & 7.7& high& 1\\ 1556367221& 27-\mathrm{Apr}-19& 05:13:41 & 4.05& low& 1\\ 1556367225& 27-\mathrm{Apr}-19& 13:3:45& 2.75& low& 1\end{array}\right]$$

In a similar manner matrix vector representations are formulated for IoT garage activity, IoT GPS_tracker activity, IoT Modbus activity including, IoT Motion_Light, IoT Thermostat activity and IoT Weather activity separately. For performing simulations, the service profile corresponding to IoT_Fridge is analyzed. With the above matrix representation, by applying min–max normalization scaling function, the maximum values (i.e., from fridge_temperature) are scaled and the resultant matrix is obtained as given below.

$$NIF=\left[\begin{array}{cccccc}1556245200& 25-\mathrm{Apr}-19& 19:20:00 & 11.55& high& 1\\ 1556245205& 25-\mathrm{Apr}-19& 19:20:05 & 1.75& low& 1\\ 1556459978& 28-\mathrm{Apr}-19& 06:59:38 & 3.2& low& 1\\ 1556459983& 28-\mathrm{Apr}-19& 06:59:43 & 4& low& 1\\ 1556459988& 28-\mathrm{Apr}-19& 06:59:48 & 4.65& low& 1\\ 1556209441& 25-\mathrm{Apr}-19& 09:24:01 & 8.65& high& 1\\ 1556209442& 25-\mathrm{Apr}-19& 09:24:02 & 9.1& high& 1\\ 1554061012& 31-\mathrm{Mar}-19& 12:36:52 & 13.1& high& 0\\ 1554061013& 31-\mathrm{Mar}-19& 12:36:53 & 8.65& high& 0\\ 1554061014& 31-\mathrm{Mar}-19& 12:36:54 & 2& low& 0\\ 1556327188& 26-\mathrm{Apr}-19& 18:06:28& 4.95& low& 1\\ 1556327189& 26-\mathrm{Apr}-19& 18:06:29& 3& low& 1\\ 1556448879& 28-\mathrm{Apr}-19& 03:54:39 & 4& low& 1\\ 1556448884& 28-\mathrm{Apr}-19& 03:54:44 & 1& low& 1\\ 1556448889& 28-\mathrm{Apr}-19& 03:54:49 & 7.7& high& 1\\ 1556367221& 27-\mathrm{Apr}-19& 05:13:41 & 4.05& low& 1\\ 1556367225& 27-\mathrm{Apr}-19& 13:3:45& 2.75& low& 1\end{array}\right]$$

The processed IoT data points are centered in such a manner so as to subtract off the mean of each column, therefore making a smooth transformation between service profiles, modeling according to distinct service profiles. Followed by which the test from observations ‘${O}_{i}$’ and ‘${O}_{k}$’ is based on log likelihood ratio is formulated as given below for IoT_Fridge service profile. In a similar manner for distinct service profiles, based on log likelihood ratio results are obtained. Then, the progressive covariance matrix is formulated. Then, with the progressive covariance matrix results, the covariance matrix results in negative representation are considered as less than ‘$\eta$’ and hence is rejected. The final extracted features are listed (for service profile: IoT_Fridge). Table 2 provides the results of log likelihood ratio, progressive covariance matrix and finally the extracted features.

**Table 2:** Tabulation results of log likelihood ratio, progressive covariance matrix and finally the extracted features.

	Results of log likelihood ratio	Results of progressive covariance matrix	Features extracted
1	${O}_{1}=\left[\mathrm{ts}\right];{O}_{2}=\left[\mathrm{date}\right]; {O}_{3}=\left[\mathrm{time}\right]; {O}_{4}=\left[\mathrm{fridge}\_\mathrm{temperature}\right]; {O}_{5}=\left[\mathrm{tempreature}\_\mathrm{condition}\right]; {O}_{6}=\left[\mathrm{label}\right]$	$Cov\left({O}_{1}\right)\left\{w.r.to 2\right\}=-49.619, Cov\left({O}_{2}\right)\left\{w.r.to3\right\}=-3.436, Cov\left({O}_{3}\right)\left\{w.r.to 4\right\}=-2.087, Cov\left({O}_{4}\right)\left\{w.r.to 5\right\}=3.135, Cov\left({O}_{5}\right)\left\{w.r.to 6\right\}=2.155,Cov\left({O}_{6}\right)=2.155$	${O}_{4}=\left[\mathrm{fridge}\_\mathrm{temperature}\right]; {O}_{5}=\left[\mathrm{tempreature}\_\mathrm{condition}\right]; {O}_{6}=\left[\mathrm{label}\right]$

$$FE\left[FV\right]=\left[\begin{array}{ccc}11.55& high& 1\\ 1.75& low& 1\\ 3.2& low& 1\\ 4& low& 1\\ 4.65& low& 1\\ 8.65& high& 1\\ 9.1& high& 1\\ 13.1& high& 0\\ 8.65& high& 0\\ 2& low& 0\\ 4.95& low& 1\\ 3& low& 1\\ 4& low& 1\\ 1& low& 1\\ 7.7& high& 1\\ 4.05& low& 1\\ 2.75& low& 1\end{array}\right]$$

Finally, based on the conditions, the detection of IIoT attack or not are evaluated according to the results in the action ‘$Act$’. Also, dynamic rewards are provided by the agent. If ‘$Act=0$’, then, normal traffic [fridge_temperature, temp_condition, label]: 13.1, high, 0; 8.65, high, 0; 2, low, 0 and the other network samples (as given below) are attack instances. Table 3 clearly explains the result attack instances and normal instances.

**Table 3:** Results of the action.

S. no.	Attack instances	Normal instances
1	$$Attack(instances)=\left[\begin{array}{ccc}11.55& high& 1\\ 1.75& low& 1\\ 3.2& low& 1\\ 4& low& 1\\ 4.65& low& 1\\ 8.65& high& 1\\ 9.1& high& 1\\ 4.95& low& 1\\ 3& low& 1\\ 4& low& 1\\ 1& low& 1\\ 7.7& high& 1\\ 4.05& low& 1\\ 2.75& low& 1\end{array}\right]$$	$$Normal\left(instances\right)=\left[\begin{array}{ccc}13.1& high& 0\\ 8.65& high& 0\\ 2& low& 0\end{array}\right]$$

### Quantitative analysis of SPC–DRRL

In this section, the quantitative analysis of sliding principal component and dynamic reward reinforcement Learning (SPC–DRRL) method is validated in terms of four metrics, namely, IIoT attack detection time, IIoT attack detection accuracy, IIoT attack detection overhead and IIoT attack detection error rate. To perform fair comparison similar numbers of network samples are utilized for validation using the four methods, SPC–DRRL, Deep LSTM AE^1^, HDRaNN^2^ and state-of-the-art method, Machine Learning^3^ respectively.

### Performance analysis of IIoT attack detection time

In this section the performance analysis of IIoT attack detection time is discussed. The time taken in detection IIoT attack remains the most significance performance metrics as early the attack detection more is the overall network is said to be. The mathematical representation of IIoT attack detection time is given below.

16 $${ADT}_{IIoT}=\sum_{i=1}^{n}{NS}_{i}*Time\left[AD\right]$$

From the above Eq. (16), the IIoT attack detection time ‘${ADT}_{IIoT}$’ is obtained on the basis of network samples ‘${NS}_{i}$’ involved in the simulation and the actual time consumed in attack detection ‘$Time\left[AD\right]$’. It is measured in terms of milliseconds (ms). Table 4 lists the IIoT attack detection time results obtained using the proposed SPC–DRRL and two existing methods, methods, Deep LSTM AE^1^, HDRaNN^2^ and the state-of-the-art method, Machine learning^2^ respectively.

**Table 4:** Tabulation of IIoT Attack detection time.

Network samples	IIoT attack detection time (ms)			
	SPC–DRRL	Deep LSTM AE	HDRaNN	Machine learning
2500	875	1225	1350	1625
5000	935	1285	1415	1735
7500	985	1315	1535	1855
10,000	1055	1385	1725	2055
12,500	1135	1455	1835	2135
15,000	1245	1585	1955	2285
17,500	1355	1725	2055	2455
20,000	1525	1835	2155	2585
22,500	1785	2055	2355	2635
25,000	2035	2255	2585	2755

**Figure 5:**
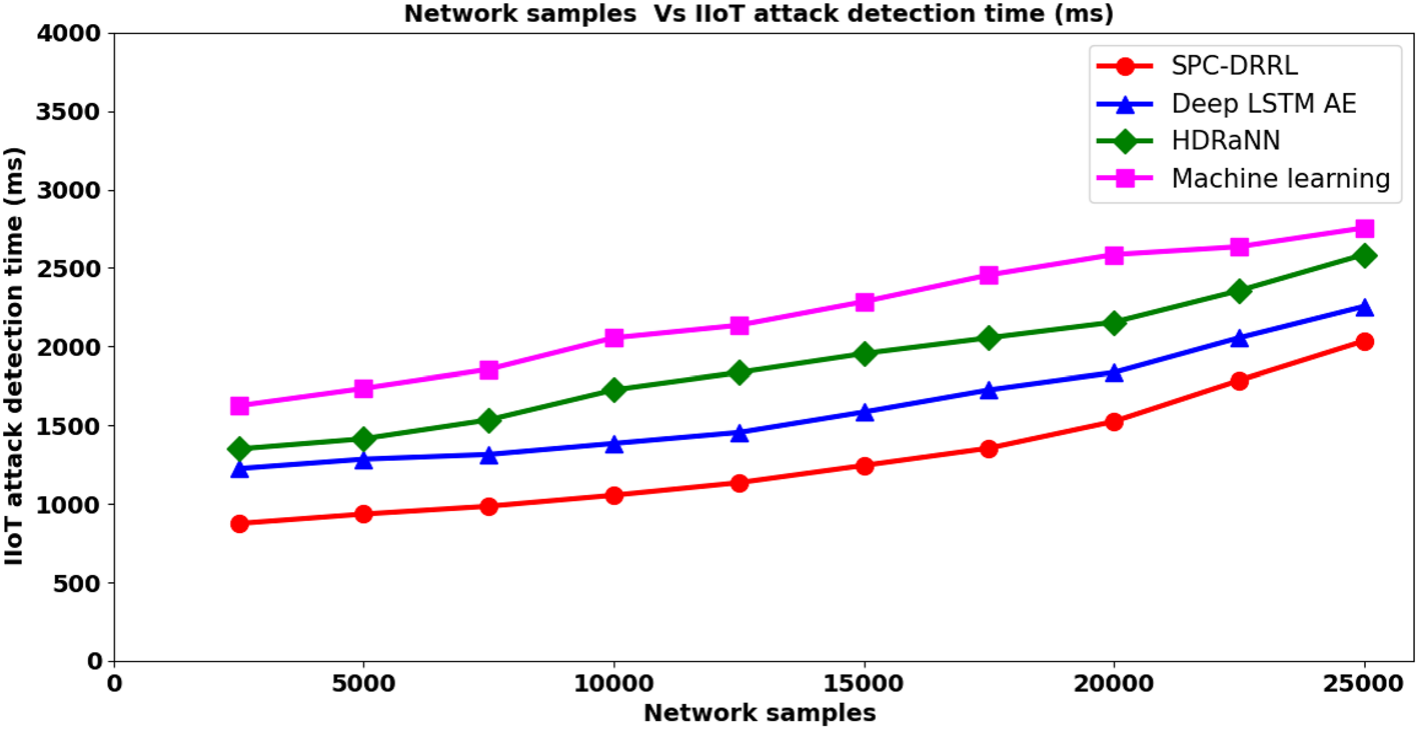
Comparative analysis of IIoT attack detection time.

Figure 5 given above shows the graphical portrayal of IIoT attack detection time using the four methods, SPC–DRRL,^1–3^. From the figure it is inferred that the attack detection time increases with the number of network samples. This is because of the reason that with larger number of network samples placed in the IIoT network, the time involved in detecting the attack also increases. So a direct proportionality is observed between the x and y axis. However, with simulations conducted using 2500 numbers of network samples, construct a reliable attack detection system, the time consumed in detecting correct attack for a particular network sample being ‘0.35*ms*’, the overall attack detection time using SPC–DRRL was 875ms, the time consumed in detecting correct attack for a particular network sample being ‘0.49*ms*’, the overall attack detection time using^1^ was 1225ms, the time consumed in detecting correct attack for a particular network sample being ‘0.54*ms*’, the overall attack detection time using^2^ was 1350ms and finally observed to be 1625ms using^2^. From this result it is inferred that the attack detection time in detecting different IIoT attacks using SPC–DRRL is better than when compared to^1–3^. The improvement is due to the application of log likelihood sliding principal component-based feature extraction algorithm in SPC–DRRL method. By applying this algorithm, initially, the normalized and scaled results were provided as input. Second, transformation between service profiles was performed employing the log likelihood ratio and finally, for each service profiles progressive covariance matrix is formulated. With this function, pertinent and essential features were extracted, therefore reducing the dimensionality and the attack detection time using SPC–DRRL by 21% compared to^1^, 33% compared to^2^ and 42% compared to^3^ respectively.

### Performance analysis of IIoT attack detection accuracy

In this section the performance analysis of IIoT attack detection accuracy is evaluated. The efficiency of the method is said to be validated based on the accurate attack detection being made by the method being designed. The mathematical representation of IIoT attack detection accuracy is given below.

17 $${ADA}_{IIoT}=\sum_{i=1}^{n}\frac{{NS}_{AD}}{{NS}_{i}}$$

From the above Eq. (17), the IIoT attack detection accuracy ‘${ADA}_{IIoT}$’ is measured based on the network samples ‘${NS}_{i}$’ involved in the simulation and the network samples accurately detected ‘${NS}_{AD}$’. It is measured in terms of percentage (%). Table 5 lists the IIoT attack detection accuracy results obtained using the proposed SPC–DRRL and two existing methods, methods, Deep LSTM AE^1^, HDRaNN^2^ and the state-of-the-art method, Machine learning^2^ respectively.

**Table 5:** Tabulation of IIoT Attack detection accuracy.

Network samples	IIoT attack detection accuracy (%)			
	SPC–DRRL	Deep LSTM AE	HDRaNN	Machine learning
2500	97.4	96.2	95.4	92.2
5000	96.35	92.35	90.25	89
7500	96	92	91	88.35
10,000	94.25	90.15	89	86
12,500	94.15	89.35	87	85
15,000	93.55	87	85	84
17,500	92.15	85.25	84.35	83.15
20,000	92	83	82	80
22,500	91.85	81	80	78.35
25,000	91	80	78	76

**Figure 6:**
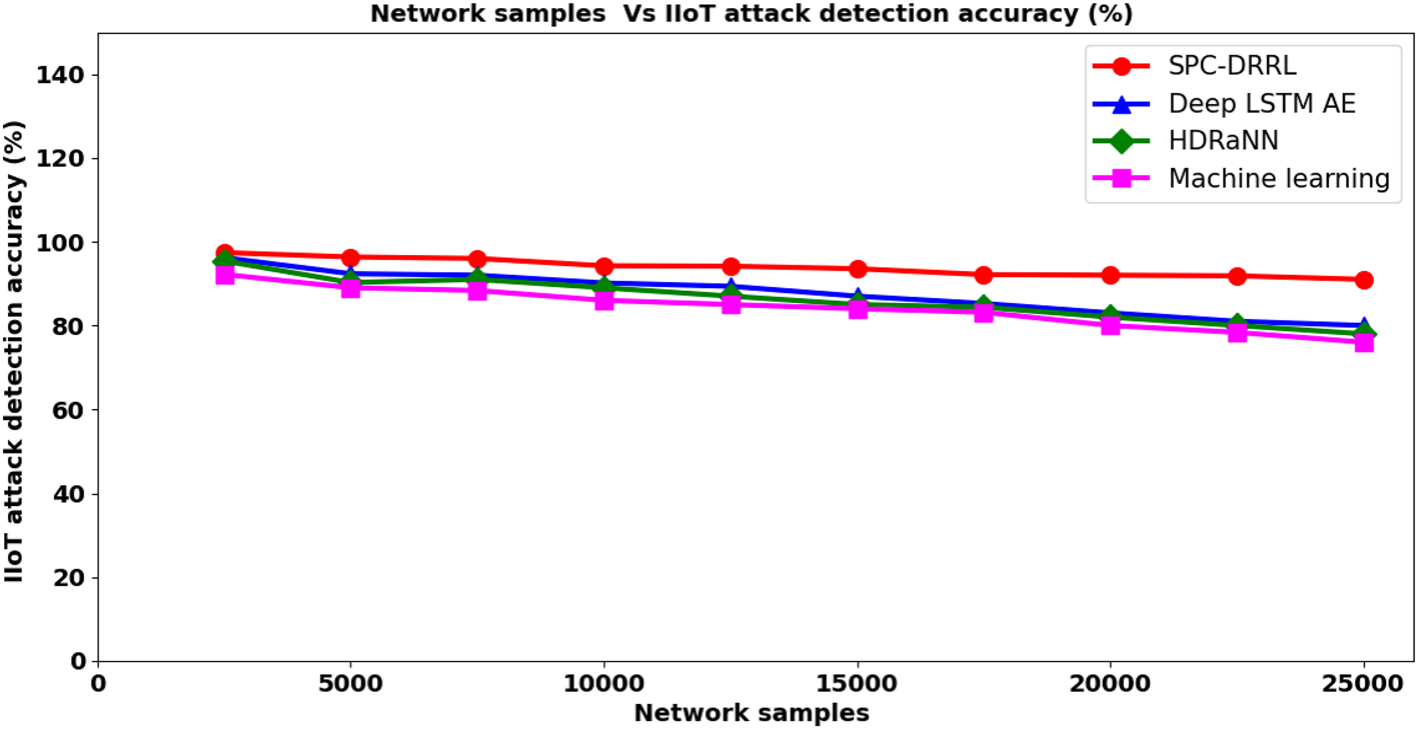
Comparative analysis of IIoT attack detection accuracy.

Figure 6 given above graphically compares the proposed SPC–DRR^1–3^ on TON_IoT dataset in terms of attack detection accuracy. In figure, X coordinates indicates network samples and Y coordinates indicates the measure of attack detection accuracy. The network samples is defined as the IoT features of different services profiles and used for experimental purpose so that attack detection made by network in terms of attack detection accuracy be measured. The reported result from figure shows that the proposed SPC–DRRL method outperforms other methods^1–3^ compared from 7%, 9% and 12% in term of attack detection accuracy. This is evident from the simulation with 25000 network samples involved in attack detection system and ‘2435’ number of network samples were correctly detected by the network using SPC–DRRL method, ‘2405’ number of network samples were detected by the network using^1^, ‘23805’ number of network samples were detected by the network using^2^ and ‘2305’ number of network samples were detected by the network using^2^. It is because SPC–DRRL method utilizes min–max normalization scaling function that eliminates the values of each feature within an explicit range, therefore ensuring attack detection accuracy into SPC–DRRL method.

### Performance analysis of IIoT attack detection error rate

The third parameter of significance is the error involved during the IIoT attack detection. This is because using this parameter also the significance of the proposed method are also said to be validated. Lower the error rate more significant is the proposed method said to be. The IIoT attack detection error rate is mathematically represented as given below

18 $${ADER}_{IIoT}=\sum_{i=1}^{n}\frac{{NS}_{WD}}{{NS}_{i}}*100$$

From the above Eq. (18), the IIoT attack detection error rate ‘${ADER}_{IIoT}$’ is measured based on the network samples considered for simulation purpose ‘${NS}_{i}$’ and the network samples wrongly detected ‘${NS}_{WD}$’ with attacks though found to be not. It is measured in terms of percentage (%). Table 6 given below provides the IIoT attack detection error rate using the proposed SPC–DRRL and two existing methods, methods, Deep LSTM AE^1^, HDRaNN^2^ and the state-of-the-art method, Machine learning respectively.

**Table 6:** Tabulation of IIoT attack error rate.

Network samples	IIoT attack error rate (%)			
	SPC–DRRL	Deep LSTM AE	HDRaNN	Machine learning
2500	1.4	1.8	2.12	2.4
5000	1.6	2.2	2.45	2.65
7500	1.8	2.35	3	3.35
10,000	2.2	2.75	3.35	4.15
12,500	2.35	3	3.85	4.55
15,000	2.55	3.25	4	5
17,500	2.8	3.55	4.15	5.35
20,000	3.1	4	4.35	5.85
22,500	3.35	4.25	4.85	6
25,000	3.85	4.45	5	6.35

Figure given above shows the impact of IIoT attack detection error rate for different numbers of network samples ranging between 2500 and 25,000 obtained at different time intervals. From the figure it is inferred that the attack detection error rate is directly proportional to the number of network samples considered for simulation. This is because of the reason that with different number of network samples obtained over a period of time in IIoT, an increasing trend is said to be observed when evaluating attack detection error rate. However, with the simulations conducted using 2500 network samples and the network samples wrongly predicted using SPC–DRRL^1, 2^ being ‘35’, ‘45’, ‘53’ and ‘60’, the overall attack detection error rate were observed to be ‘1.4%’, ‘1.8%’, ‘2.12%’ and ‘2.4%’ respectively. From these results it is inferred that the attack detection error rate is lesser using SPC–DRRL when compared to^1–3^. The results behind is due to the application of Dynamic Reward Reinforcement Learning-based IIoT attack detection model. By applying this model, incident repository is employed that stores the intermediate and final action results. Therefore by looking into this incident repository, network samples being attack are discarded during further processing and only the network samples to be of genuine is considered for further processing. With these factors, the IIoT attack detection error rate using SPC–DRRL is said to be reduced by 21% compared to^1^, 33% compared to^2^ and 45% compared to^3^ respectively.

### Performance analysis of IIoT attack detection overhead

Finally, in this section IIoT attack detection overhead is discussed. A small portion of overhead is said to be equipped while performing the attack detection process and this is said to be IIoT attack detection overhead. The mathematical formulate of IIoT attack detection overhead is given as below.

19 $${ADO}_{IIoT}=\sum_{i=1}^{n}{NS}_{i}*Mem\left[Act\right]$$

From the above Eq. (19), the IIoT attack detection overhead is measured ‘${ADO}_{IIoT}$’ based on the network samples ‘${NS}_{i}$’ and the memory consumed ‘$Mem\left[Act\right]$’ in detecting attack. It is measured in terms of kilobyte (KB). Table 7 given below list the IIoT attack detection overhead using the proposed SPC–DRRL and two existing methods, methods, Deep LSTM AE, HDRaNNand the state-of-the-art method, Machine learning respectively.

**Table 7:** Tabulation of IIoT attack detection overhead.

Network samples	IIoT attack detection overhead (KB)			
	SPC–DRRL	Deep LSTM AE	HDRaNN	Machine learning
2500	625	775	975	1125
5000	675	825	1025	1185
7500	735	955	1135	1235
10,000	825	1035	1255	1315
12,500	1035	1155	1455	1535
15,000	1125	1355	1835	2035
17,500	1315	1525	2055	2155
20,000	1435	1735	2135	2535
22,500	1525	1955	2255	2725
25,000	1825	2035	2435	2915

**Figure 7:**
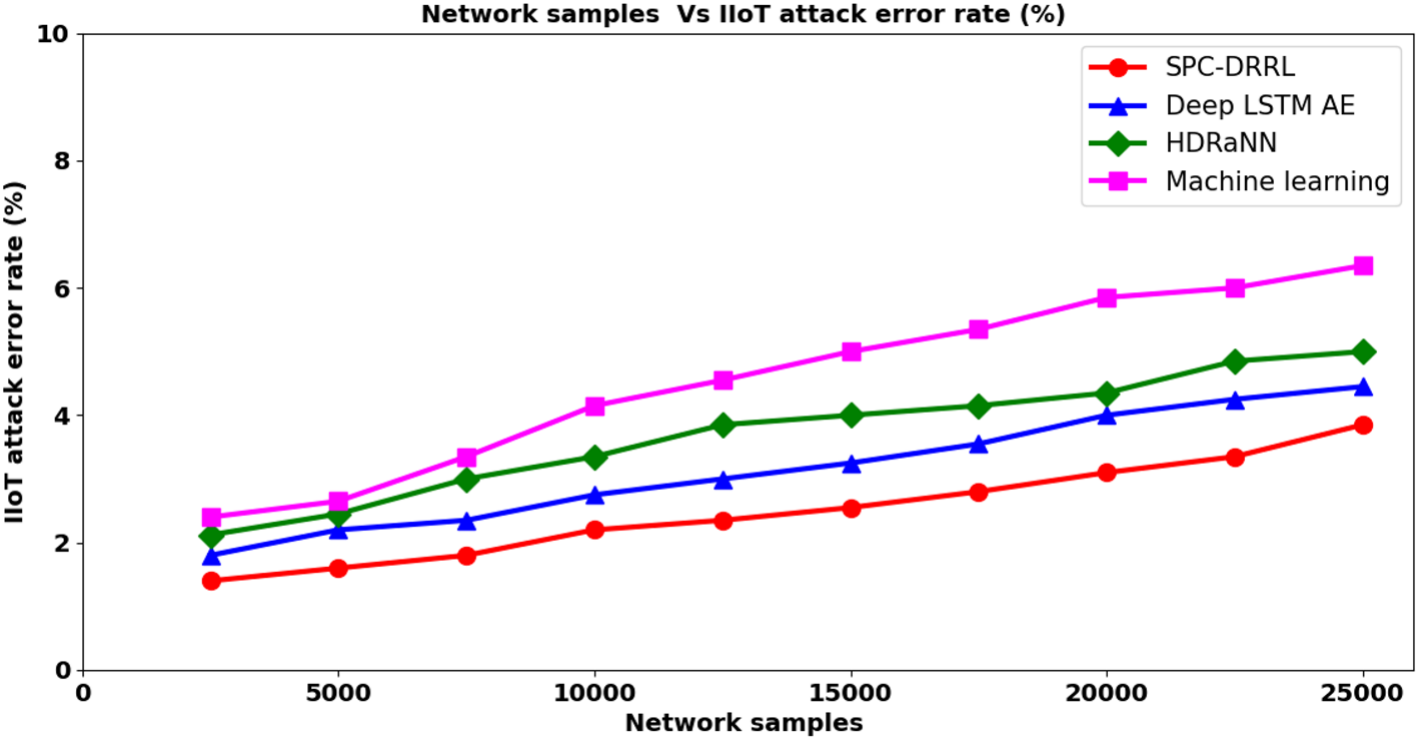
Comparative analysis of IIoT attack detection error rate.

**Figure 8:**
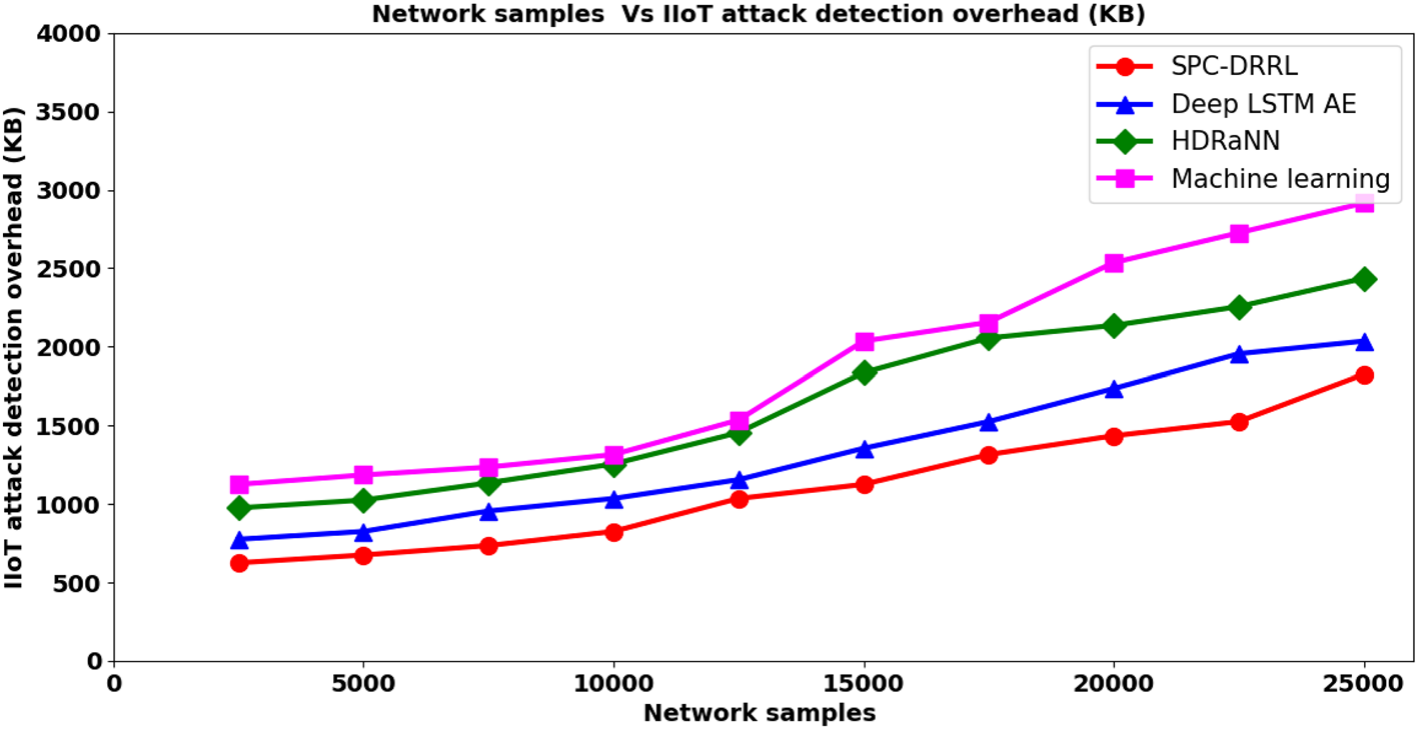
Comparative analysis of IIoT attack detection overhead.

Finally, Fig. 7 given above illustrates the IIoT attack detection overhead with respect to 25,000 distinct network samples conducted with an average of 10 simulation runs using SPC–DRRL^1–3^. Figure 8 represents the comparative analysis of IIoT attack detection overhead. The attack detection overhead is also found to be increasing with the increasing numbers of network samples. This is obviously due to the reason that with the increase in the network sample size results in congestion and also the action results that has to be stored in the incident repository also gets increased. This in turn would increase the attack detection overhead also. However, the comparative analysis showed betterment when applied with SPC–DRRL upon comparison to^1–3^. The reason was owing to the application of Dynamic Reward Reinforcement Learning-based IIoT attack detection algorithm. By applying this algorithm, employing the dynamic reward function and application of its results for obtaining the loss function results in the minimization of memory involved during the overall evaluation of action results. This in turn reduced the memory involved in attack detection also using SPC–DRRL method by 17% compared to^1^, 33% compared to^2^ and 41% compared to^3^ respectively.

## Conclusion

In many IIoT attack detection systems, the similarity scores at a fine grained manner are usually utilized. In compared to most of the prevailing IIoT attack detection methods, a novel sliding principal component and dynamic reward reinforcement learning (SPC–DRRL) using deep reinforcement learning based on network samples is proposed to improve the detection accuracy in addition to minimizing the time and error rate is proposed in this article. The main innovation of our method is obtaining a measure for different service profiles (i.e., involving different activities) by proposing log likelihood sliding principal component-based feature extraction algorithm. Specifically, an input feature vector matrix is first created and measured using the log likelihood ratio to measure the likelihood of obtaining the principal component in a specific sliding window. Here, each service profiles are said to be performed in each sliding window. Second, the Dynamic Reward Reinforcement Learning-based IIoT attack detection is presented to provide detection of IIoT attacks via incident repository and generate attack detection results. In addition, along with the experiments, an empirical evaluation of our method with the aid of discussion was performed to compare to the traditional and state-of-the-art methods using the ToN_IoT dataset. The limitations of the proposed methods are IIoT systems frequently on exclusive technologies and protocols, making it complex for several systems to converse. Limit the scalability and flexibility of IIoT systems and improve the cost of implementing and maintaining IIoT systems.The observed numerical results have confirmed that the proposed SPC–DRRL method outperforms well by achieving a higher attack detection accuracy, minimum overhead and error rate than the other state-of-the-art methods.

## Data Availability

The data that support the findings of this study are available from the corresponding author, upon reasonable request.
